# Caregiver Perspectives on Barriers to Accessing Behavioral Health care: Opportunities and Challenges for Pediatric Settings

**DOI:** 10.1016/j.acap.2026.103287

**Published:** 2026-03-12

**Authors:** Maheen Ibrahimi, Selamawit Tsegaye, Janhavi Kulkarni, Marisleysis Gonzalez, Katherine Guyon-Harris

**Affiliations:** University of Pittsburgh School of Medicine (M Ibrahimi and S Tsegaye), Pittsburgh, Pa; University of Pittsburgh School of Medicine (J Kulkarni, M Gonzalez, and K Guyon-Harris), Department of Pediatrics, Pittsburgh, Pa

**Keywords:** access, behavioral health care, early intervention, pediatrics, primary care

## Abstract

**Objective:**

Families with young children have regular, frequent interactions with pediatric primary care across early childhood. Thus, pediatric primary care is an ideal setting to prevent mental and behavioral health disorders through supporting access to behavioral health care services. Exploring the barriers families with young children face in accessing behavioral health care services will support insights into potential strategies to improve access through primary care settings.

**Methods:**

Semi-structured qualitative interviews were used to explore the perspectives of 27 caregivers of young children (ie, ages 0–5 years) regarding access to behavioral health care. Participants were recruited from a large pediatric clinic affiliated with a pediatric residency program serving a high proportion of Medicaid-eligible families (> 85%). Deductive and inductive methods were used to develop a codebook followed by content analysis to identify and characterize access barriers.

**Results:**

Qualitative content analysis revealed several interconnected barriers families face accessing behavioral health care services for their young children. Barriers were organized into the categories of logistical, financial, and psychosocial. Logistical barriers included 1) childcare, 2) time, 3) transportation, and 4) employment-related issues. Financial barriers included 1) inadequate insurance coverage, and 2) broader financial constraints. Psychosocial barriers included 1) education and understanding of behavioral health issues, 2) anxiety or shame, 3) judgment, and 4) mistrust of health care providers.

**Conclusions:**

Improving access to behavioral health care services through pediatric primary care settings will require a multifaceted approach that addresses logistical, financial, and psychosocial barriers. Solutions should consider systemic and individual factors, promote behavioral health education, and reduce stigma.

In the United States, nearly 1 in 5 children meet criteria for a mental health disorder, with concerns often raised during early childhood through well-child pediatric visits.^1^ Early identification and treatment of behavioral health challenges in infancy and toddlerhood can prevent progression into mental and behavioral health disorders later in childhood.^2–4^ Behavioral health challenges for very young children can include difficulties with emotional and behavioral regulation (eg, excessive tantrums, aggression, fears and worries, sleep and feeding issues) as well as parent-child relationship issues. Despite the importance of early intervention, many children fail to establish timely contact with behavioral health treatment, particularly those with early-onset mood and anxiety disorders.^5^ Young children cannot access care on their own; the experiences of caregivers in accessing behavioral health care are an important issue.

Pediatric behavioral health access issues may be caused by barriers, including logistical and financial restraints, limited provider availability, and systemic challenges such as fragmented care, with children from low-income households disproportionately affected.^6–9^ Stigma, fears of overmedication, and concern about punitive responses, such as Child Protective Services, may further discourage families from seeking care.^7^ Pediatric primary care has been increasingly recognized as a promising setting for improving behavioral health access for children, especially when behavioral health services are integrated within the medical home.^10,11^ Systematic reviews have demonstrated the feasibility of preventative behavioral health programs in primary care, with evidence that these models can improve early identification and intervention.^12,13^ Furthermore, parents report high interest in primary-care-based behavioral services, citing convenience, trust in pediatric providers, and reduced stigma compared to specialty settings.^14–17^ Provider perspectives further underscore the potential of integrated approaches but highlight challenges related to training, time constraints, and lack of external resources.^18–22^ Structural barriers such as insurance policies, location of services, and the separation of physical and behavioral health care systems further exacerbate these concerns.^8^

Existing literature on barriers to behavioral health services primarily focuses on adults or adolescents, overlooking the unique challenges families with young children encounter, especially from the perspectives of their caregivers. Furthermore, caregivers play an essential role in navigating behavioral health care systems for young children, yet their perspectives on what prevents or facilitates access remain underexplored. This study bridges gaps in previous literature by examining caregiver perspectives on barriers to accessing behavioral health care with a focus on young children to inform strategies to promote equitable access to behavioral interventions in pediatric settings.

## Methods

### Participants

Participants for the present study were recruited from a pediatric clinic affiliated with a large academic hospital system and pediatric residency program. Caregivers were eligible to participate if they were caring for at least 1 child aged 0 to 5 years who was a patient at the clinic. The clinic is situated in an urban area and serves a high proportion of Medicaid-eligible families (> 85%).

### Procedures

This qualitative study utilized semi-structured 1-on-1 virtual interviews, approximately 30 to 45 minutes long, to explore the perspectives of families with young children. Purposive sampling was used to approach caregivers with young children in the waiting room presenting for a pediatric appointment. Caregivers completed an online survey that began with the consent form and a series of eligibility questions. Eligible caregivers completed a survey that ended with a question asking if they were interested in being contacted for a follow-up interview about behavioral health care services for young children. Interested caregivers were taken to a separate survey form to leave their preferred contact information.

The interviews were administered utilizing a guide (Digital Supplement 1) developed by the senior author (a clinical psychologist [PhD]) with input from pediatric colleagues. Interviewers (third and fourth authors) were an advanced undergraduate student in public health and a bachelor’s-level research coordinator with a degree in psychology who were trained and supervised by the senior author. Interviewers were also recruiters for the study and had briefly met the participants prior to the interview. Informed consent was obtained, along with permission to audio record the interview. Interview questions focused on participants’ thoughts and experiences related to accessing behavioral health care broadly and for their own children, with demographic information collected at the end of each interview. Participants were compensated for their time with a $30 gift card.

The interviews were transcribed verbatim and checked for accuracy between 2 transcribers on a team of pre- and post-bachelor’s research assistants in psychology and public health. A codebook was developed by the senior author and first and second authors (medical students interested in pediatrics) using both deductive and inductive methods. From a deductive perspective, we had an a priori interest in coding for mentions of barriers to access to behavioral health care and created codes around this topic. From an inductive perspective, we also allowed for the elucidation of new codes that were not planned a priori. The first and second authors, with support from the senior author, then coded all interviews utilizing the program Nvivo. Transcripts were double-coded and fully adjudicated, with the senior author serving as the final judge as needed.

### Data Analysis

A qualitative analysis was conducted in a content analysis framework to reduce text to identify and organize core concepts.^23^ The lead author conducted the content analysis to identify core consistencies in barriers to behavioral health care access and organize barriers into categories to facilitate interpretation.

## Results

### Participant Characteristics

Sixty-eight survey participants indicated that they were interested in a follow-up interview, and we attempted outreach in order of survey receipt until thematic saturation.^1^ We attempted to reach 42 caregivers of which 27 responded and completed an interview. The remaining 15 either did not respond (n = 12) or were reached but did not complete an interview (n = 3; eg, no show or cancellation without rescheduling). The final sample included 27 caregivers (M age = 30.7, SD = 6.3) of at least 1 child under the age of 6 (Table 1). Eleven percent of caregivers identified as Hispanic. Seventy percent identified as African American and 30% as white. Forty-four percent reported an income below 40k and 48% were employed full-time. Our sample was representative of the larger clinic which serves a population of families that are greater than 85% Medicaid eligible and predominantly identify as African American.

### Content Analysis Findings

Several interconnected barriers that families face when accessing behavioral health care for their young children were categorized into logistical, financial, and psychosocial challenges (see Table 2 for further description of barriers and exemplar quotes). Logistical barriers included 1) childcare, 2) time, 3) transportation, and 4) employment-related issues. Financial barriers were evident in the form of 1) inadequate insurance coverage, and 2) broader financial constraints that made affording care difficult. Psychosocial barriers included 1) education and understanding of behavioral health issues, 2) anxiety or shame, 3) judgment from others, and 4) mistrust in health care providers.

### Logistical Barriers

Logistical constraints were frequently cited among participants. A prominent theme was the lack of accessible, affordable childcare. Parents emphasized the difficulty of coordinating care for one child while also juggling multiple caregiving responsibilities. One participant stated, “It’s a issue for me specifically because I would either have to miss work or pay somebody to babysit my children to get mental health services” (Caregiver 8). This sentiment illustrates how intertwined caregiving responsibilities are with seeking care, as many behavioral health appointments require not just time, but space, free from distractions and additional dependents.

Time was another barrier, particularly for working parents. Families described an overwhelming sense of being stretched too thin, unable to fit therapy or assessments into the workday. One caregiver explained, “A lot of parents work during the day and then when they get home, they don’t really have the time to engage in therapy all the time” (Caregiver 17). Another parent shared that “it’s been difficult times in immediate care” (Caregiver 11), referencing the long wait times for appointments and limited flexibility in scheduling.

Transportation also emerged as a significant barrier. For many, simply getting to an appointment, especially with children, proved extremely difficult. “Without the transportation, you can’t get to wherever you need to go to get those services” (Caregiver 16), one parent said. Others pointed out that even public transportation was not always reliable, accessible, or affordable, particularly in areas not well-served by public transit.

Employment-related barriers further compounded the problem. Several participants discussed jobs that lacked the flexibility to take time off. Others described employers who were unsupportive of caregiving demands. “You can’t really miss out on your job because…your job’s more important than basically, your mental health” (Caregiver 6), one caregiver shared, highlighting how strict work conditions limited their ability to prioritize their child’s health.

### Financial Barriers

For many caregivers, the cost of behavioral health services posed a significant barrier. Even when families had health insurance, coverage was often described as inadequate, confusing, or conditional. Parents expressed frustration with which services were covered, saying that they frequently encountered unexpected costs. One parent explained, “Some insurances won’t cover it. They won’t cover therapy, it might come out of pocket or it only cover partial of it” (Caregiver 22). Another elaborated that “if the insurance doesn’t cover it, then they won’t even be able to like sign up and be a part of a group and actually get help” (Caregiver 7), showing how coverage restrictions often determined whether families could even pursue care.

Additionally, caregivers shared that copays, paperwork issues, or unexpected denials created further financial strain. One participant recalled, “They’ll be able to take the insurance but there’s also a copay fee or there’s something wrong with the insurance and it’s just a struggle” (Caregiver 6). This led many to avoid services altogether or delay care for having to make trade-offs between health care and other necessities.

These struggles were compounded by broader financial hardship. Many caregivers were either underemployed or relying on public assistance. The stress of navigating daily financial survival left little room for “optional” expenses, even when families knew behavioral health services were necessary. As one parent explained, “Everyone don’t have a job and it’s hard to pay bills and stuff with money through just welfare or something” (Caregiver 4). Thus, behavioral health care may be deprioritized in favor of more immediate needs like rent, food, or utilities.

### Psychosocial Barriers

Beyond logistical and financial constraints, caregivers identified a range of psychosocial challenges that impacted their ability or willingness to access behavioral health services. One was a lack of awareness or understanding of what behavioral health is, how it can help, or where to find services. Several parents described feeling lost in a system they didn’t know how to navigate. One said, “People don’t know that there is like known knowledge about that. They don’t know that they can reach someone that can help them” (Caregiver 26), while another noted, “…a lot of people don’t know how to find stuff or how… certain services are out there” (Caregiver 9). This lack of knowledge often served as a powerful barrier, particularly for families without previous exposure to mental health systems.

Caregivers also described experiencing internal psychological barriers, such as anxiety, shame, and fear of failure that sometimes prevented them from seeking care or caused them to second-guess their decision. As one participant explained, “They can wanna take the step forward to do [therapy] but then when it comes down to that they can have bad anxiety or just have issues with tackling that situation” (Caregiver 16). Others worried that acknowledging their child’s struggles reflected poorly on their parenting. “There’s still a lot of stigma involved with getting—seeking out therapy… it’s that feeling of you know oh I failed as a parent” (Caregiver 17), one caregiver shared.

Judgment from others—especially family, community members, or even providers—was also a significant concern. Parents worried about being perceived as weak, unfit, or overreacting. One participant said, “Some people are judgmental and instead of judging, they should just be understanding. And it’s hard for people with mental health problems to even trust people as it is” (Caregiver 5). Another added, “There are a lot of parents that are trying and doing their best and I think that… judgment is a major thing” (Caregiver 21).

Mistrust of health care providers was also a significant theme. Some caregivers felt that providers did not genuinely care about their children or had ulterior motives. “They’re [primary care providers] just in it cause of the money and it pays the bills like people don’t care to take their jobs seriously” (Caregiver 10), one parent shared. Others feared that seeking help could backfire, leading to reports to child protective services or punitive outcomes: “they would rather call child protective services instead of offering like additional help for the parent… that just scares the parent away more” (Caregiver 8). Overall, psychosocial barriers, ranging from internal doubts to external judgment, served as powerful deterrents, shaping how families understood behavioral health care and whether they felt safe or justified in seeking it.

## Discussion

This qualitative study explored the perspectives of caregivers with young children regarding behavioral health care access and the barriers they have experienced. We identified 3 main categories of barriers: logistical, financial, and psychosocial—each divided into subcategories that further characterized specific aspects that families felt impacted access to behavioral health care for their children.

Logistical barriers such as lack of childcare, time constraints, transportation difficulties, and employment-related conflicts were common across participants. These findings are consistent with existing research that points to the burdens of coordinating behavioral health appointments alongside work and caregiving responsibilities, particularly for families with limited support systems.^7,8^ Prior studies of parents in rural and urban settings likewise describe the challenges of aligning behavioral health care appointments with family schedules and resources.^15,17^ Unlike more structural critiques focused on workforce shortages or service availability,^24^ our participants emphasized day-to-day logistical barriers that interfered with even initial engagement with services.

Interestingly, although Blais et al^25^ reported widespread concern about behavioral health provider shortages, our participants did not directly identify this as a barrier. Instead, they frequently described not knowing where to go or how to access services, suggesting that provider shortages may influence access indirectly by limited outreach, visibility, and entry points to care. Prior work has shown that families value pediatric primary care as a familiar and trusted place to address behavioral health concerns, given that it eliminates the need to navigate unfamiliar systems.^14,17^ Thus, situating behavioral health care services within pediatric settings could capitalize on the trust and familiarity of such settings while reducing barriers of not knowing where to go or how to access services. However, recent systematic reviews have noted that trust in primary care may not be a fruitful mechanism to support engagement among other challenges, including unknown long-term benefit of co-located care and lack of implementation outcomes which could impact systems’ willingness to try this new model.^12,13^ Furthermore, there are potentially important nuances between simply co-locating behavioral health care compared to truly embedded or integrated programs that are not just co-located but infused into the pediatric care setting. Future work is needed to further understand the benefits and challenges of co-located and embedded care models.

Policy-related barriers were less directly identified. Toure et al^26^ identified policy restrictions, such as state directives prohibiting trauma screening or lack of school-based behavioral health services. Our participants did not explicitly name such policies, but described experiences that could reflect the downstream effects of restrictive policies, such as missed appointments, difficulty obtaining referrals, or disruptions in care continuity without identifying these as systemic or policy-driven issues. This disconnect suggests that while families may be affected by policy-level barriers, these often remain invisible as they shape access to care.

Financial barriers also significantly impacted families’ ability to seek care. While many participants had health insurance, they frequently described it as inadequate or confusing, with limited behavioral health coverage and unexpected out-of-pocket costs. Prior work has shown that private insurance often fails to provide comprehensive coverage of behavioral health for low-income families^27^ and aligns with parent reports that cost is among the most decisive factors in whether families pursue services.^16^ One insurance-related challenge is the requirement of a diagnosis to receive reimbursement for behavioral health care services, which can be challenging for very young children where formal (CPT code) diagnoses either do not exist or providers are hesitant to assign diagnoses at such a young age. Insurance companies could improve access to behavioral health care by allowing for greater flexibility in diagnosis requirements or the codes that can be used for reimbursement. Furthermore, acknowledging and supporting the importance of preventive behavioral health services for young children, such as parenting and parent-child intervention programs (which are more traditionally offered through home visiting), may be one avenue for improving insurance coverage for behavioral health care visits.

Psychosocial barriers were among the most salient in our findings. Caregivers reported uncertainty about behavioral health, discomfort with labeling their child’s needs, and fear of judgment from family, community members, or health care providers. These internal and interpersonal concerns are consistent with broader literature on stigma and parental ambivalence in seeking mental health services for young children.^28,29^ Additionally, several participants reported mistrust in the health care system, including fear of punitive consequences (such as involvement of child protective services), echoing findings from prior studies that highlight how historical trauma, discrimination, and negative past experiences can erode trust in health care institutions.^30,31^ This is particularly important in communities of color and low-income populations, who are often misdiagnosed and less likely to receive timely behavioral health care.^32–34^

Our findings also align with the Aday and Andersen Behavioral Model of Health Services Use,^35^ which organizes barriers to care into predisposing, enabling, and need-based factors. Caregivers in our study described a range of predisposing factors, including health misconceptions, anxiety, and mistrust. Enabling factors were logistical restraints, childcare demands, and insurance limitations. Finally, need-based factors included recognition of their child’s socioemotional challenges. This framework provides a helpful lens to interpret and address the multilevel challenges identified by families and suggests that interventions must operate at each of these levels to meaningfully improve access.

The results of this study highlight the need for a holistic approach to improving access that considers both systemic and individual factors that limit access to behavioral health care for families with young children. Strategies to address these barriers may include improving insurance coverage and financial support, expanding or increasing access (eg, clinics providing bus fare) to public transportation options, and offering flexible appointment scheduling to accommodate diverse family needs. Public education initiatives aimed at increasing awareness and reducing stigma can also foster a more supportive environment for families. By coordinating efforts that address these barriers collectively, significant improvements in behavioral health care access for young children could be made, ultimately promoting better long-term health outcomes.

A key aspect of this study is its focus on caregiver perspectives, rather than provider or system-level experiences. Since parents are the primary gatekeepers to their children’s health care, their insights are essential in understanding why some families may delay or avoid seeking services. Based on their responses, there is a need for parent-centered solutions, such as flexible appointment scheduling to accommodate work and childcare demands, improved caregiver education to enhance awareness and trust in behavioral health services, and community-based initiatives that strengthen relationships between families and their health care providers. Interventions can therefore be more effectively modified to meet the needs of families and improve access to early pediatric behavioral health care.

### Limitations

Although efforts were made to recruit a diverse range of participants, the study’s results may not be fully generalizable to all families with young children, particularly outside of an urban clinic setting serving Medicaid-eligible families. Similarly, barriers identified by parents of young children may differ meaningfully from those experienced by parents of older children or adolescents, particularly given the intersection of behavioral and developmental concerns in early childhood. Another potential limitation is that coders were not blind to study hypotheses which may have predisposed coding towards the identification of barriers, however, coding teams were coding a broad range of codes beyond just barriers to care and met frequently to review codes and address issues of bias and drift. Finally, participants’ perspectives are shaped by their unique experiences and contexts, which may limit the broader applicability of the findings. We did not require that our participants have children with behavioral health challenges or have had experiences navigating behavioral health care settings because our goal was to understand the broad experiences and perspectives of caregivers in pediatric settings. This may have produced different results than if we had restricted enrollment to parents with such experiences.

## Conclusions

By drawing attention to the insights from families and providers, addressing systemic barriers, and implementing targeted strategies, behavioral health care services can become more accessible and impactful for young children. Further research should focus on addressing the concerns of caregivers directly by using community-based interventions and individualizing care for each family as behavioral health barriers can vary per child. Continued efforts to refine and expand these approaches will be essential in ensuring that all families, regardless of their socioeconomic background, can access the behavioral health support they need for their child.

## Supplementary Material

1

Supplementary data associated with this article can be found in the online version at doi:10.1016/j.acap.2026.103287.

## Figures and Tables

**Table 1.** Demographic Characteristics of Participants (N = 27)

Characteristic	M	SD
Age	30.74	6.273
Number of children under the age of 5	1.41	0.572
	n	%
Ethnicity		
Hispanic	3	11.1
Non-Hispanic	24	88.9
Race		
African American	19	70.4
White	8	29.6
Household Income		
Less than $39,999	12	44.4
$40,000–$99,999	8	29.6
Above $100,000	3	11.1
Unsure/Don’t know	4	14.8
Highest level of education		
High school/GED or less	10	37
Trade/technical/vocational training/	3	11.1
Associate’s degree		
Some college credit, no degree	7	25.9
College/Bachelor’s degree	3	11.1
Master’s or Doctorate Degree	4	14.8
Student status		
Yes	9	33.3
No	18	66.7
Employment status		
Employed full-time (30–40 h per week)	13	48.1
Employed part-time (less than 30 h per week)	5	18.5
Unemployed	7	25.9
Other	2	7.4

**Table 2.** Categorization of Behavioral Healthcare Barriers

Category	Subcategory	Participant Quote
Logistical	Childcare	*8: “It’s a issue for me specifically because I would either have to miss work or pay somebody to babysit my children to get mental health services.”* *23: “For the parent to get help they would need childcare.”* *3: “Maybe they didn’t have no baby, a babysitter for their other child and they wasn’t allowed to bring the other child to the— to the appointment—the other child to the appointment.”* *31: “Not having people to really help with maybe their other kids, you know, there’s a lot of things that could, you know, hinder that.”* *10: “it’s very hard when you ain’t got childcare and that was the only person that was watching all your children”*
	Time	*17: “A lot of parents work during the day and then when they get home they don’t have this really have the time to engage in therapy all the time and so it just a matter of scheduling and when those services are available if they’re not available after typical work hours or something”* *11: “I have to go in person to see my regular doctor to refill my prescription but that’s three weeks out. So it’s been difficult times in immediate care”* *34: “What can make it hard is some parents might not have the time.”* *4: “Yeah because school sometimes it’s hard to get appointments around the time kids go to school between when they get out to make it back on time”*
	Transportation	*16: “Without the transportation, you can’t get to wherever you need to go to get those services.”* *“Because some people don’t have vehicles and some people don’t have the income to even pay for a bus”* *10: “Yeah so if you don’t have um money to get transportation to get to the doctors then you know you can’t obviously go”* *9: “I don’t have a car right now. Um, yeah if you’re not mobile, then you can’t just get in the car and go. It-it can be challenging.”* *7: “Um it’d be hard because they don’t have a way to get— say they have a group or whatever or therapy. They don’t have — they can’t get there. So, that would add on to whatever stress they are already going through.”*
	Employment-related barriers	*6: “It’s very hard trying to figure out how you’re going to do things day by day, when the job’s not cooperating with you.”* *6: “It might be a challenge or a barrier because some jobs won’t cooperate with you.”* *24: “You may need to take off work so it’s not as easy to get to these places.”* *3: “Not having a job.”* *16: “Income’s a big part because you can’t really miss out on your job because for that, like, your job’s more important than basically, your mental health at that point, ‘cause you need to provide the funds to survive so you can’t really put yourself first, I would say.”*
Financial	Insurance	*22: “Some insurances won’t cover it. They won’t cover therapy, it might come out of pocket or it only cover partial of it.”* *22: “I’d say insurance might make it hard. There’s some places you can’t go because your insurance doesn’t cover it.”* *6: “ …some places you know, might not be able to take that insurance or um they’ll be able to take the insurance but there’s also a copay fee or there’s something wrong with the insurance and it’s just a struggle.”* *5: “Insurance too. You know what I mean. I feel like there should be programs you don’t need insurance.”* *7: “if the insurance doesn’t cover it, then they won’t even be able like to sign up and be a part of a group and actually get help.”* *19: “Insurance, or don’t have the money to even pay for it cause a lot of people who just because it is supposed to be covered, they just can’t get the proper needs for their children.”*
	Other	*4: “Everyone don’t have a job and it’s hard to pay bills and stuff with money through just welfare or something.”* *7: “Um cause a lot of them you have to pay for a lot of parents aren’t financially stable to pay for that.”*
Psychosocial	Education	*26: “People don’t know that there is like known knowledge about that. They don’t know that they can reach someone that can help them.”* *26: “I think that the problem is that for many people is still not considered as a important thing uh that mental health is still not uh, it’s—it’s kind of like uh you know taboo.”* *9: “…a lot of people don’t know how to find stuff or how or you know that certain services are out there”*
		*21: “And like my whole entire family is like, ‘he doesn’t need all those services, he just doesn’t wanna talk’ and like what if that’s not the case, And I waited all this time, and now he’s, he doesn’t, you know what I mean, he can’t communicate well and then he becomes aggressive”* *1: “Like the education around maybe that something’s wrong with their kid. Maybe they wouldn’t know that.”*
	Anxiety or shame	*16: “…they can wanna take the step forward to do but then when it comes down to that they can have bad anxiety or just have issues with tackling that situation.”* *17: “I think also too unfortunately I think there’s still a lot of stigma involved with getting– seeking out therapy um you know for you know parenting because then you know it’s that- that feeling of you know oh I failed as a parent”* *17: “So there’s a sort of shame that comes with that even though I don’t think there needs to be but um I know that that’s definitely something a lot of people feel is just sort of shame or feelings of failure when it goes to that.”* *26: “I would say that many of them uh would feel ashamed uh to do that because they you know, they—they maybe they think that they—they are doing fine or they know that they are not doing fine but they don’t want to expose themselves”* *16: “…maybe if they’re just like have anxiety very bad like that you’re scared to like lose—you know, put yourself out there to go in that situation. You are just scared of what to be, rather than just going for it.”* *23: “Probably I don’t wanna say afraid but you know fearful they don’t know what to expect. You know you don’t wanna feel like a failure like you don’t even know how to take care of your own kid.”* *17: “…for some people once they miss that first appointment and they’re like there’s that I don’t know like it’s almost like an apprehension or anxiety where like oh I missed the first one and now- and now I don’t know if I can go to the second one because I missed the first one and so then just it becomes a snowball effect”*
	Judgment	*5: “Some people are judgmental and instead of judging, they should just beunderstanding. And it’s hard for people with mental health problems to even trust people as it is, so the barrier with that would be having to trust someone.”* *21: “But there are a lot of parents that are trying and doing their best and I think that that is the major—like judgment is a major thing as well.”*
	Mistrust in health care providers	*10: “…they’re [primary care providers] just in it cause of the money and it pays the bills like people don’t care to take their jobs seriously and it’s just that.”* *8: “[Primary care providers] would rather call child protective services instead of offering like additional help for the parent. They immediately go into this whole other spectrum and that just scares the parent away more…”* *10: “…if they really wanna come in and help my babies like I don’t need them comin in and not helping and making the situation worse. So some– if they have to be genuine people that are really like me.”* *5: “And it’s hard for people with mental health problems that even trust people as it is so the barrier with that would be having to trust someone.”*

## What’s New

This study identifies barriers faced by families with young children in accessing behavioral healthcare, such as logistical, financial, and psychosocial challenges. The findings support integrating behavioral health services within pediatric primary care to improve access and enhance early intervention efforts.

## Footnotes

^1^ The interview team and senior author met periodically during the interview process to monitor saturation. Our survey clearly stated that not all who indicated interest would be contacted for an interview.
