# Supplementary material for: Caregiver Perspectives on Barriers to Accessing Behavioral Health care: Opportunities and Challenges for Pediatric Settings
Source: Acad Pediatr. Author manuscript; Available in PMC 2026 Jun 24. (PMC13291844; doi:10.1016/j.acap.2026.103287)
Supplement: 1 [file NIHMS2184334-supplement-1.docx]

DIGITAL SUPPLEMENT 1

FAMILIES OF YOUNG CHILDREN INTERVIEW GUIDE

Thank you for taking the time to speak with us. As I mentioned earlier, we would like to audio record this interview. Before we get started, do I have your verbal permission to begin recording?

As we begin, remember that what we talk about will not be shared with your child’s pediatrician or other providers at the Primary Care Clinic in Oakland and does not have any impact on your services at Children’s Hospital or UPMC - this is a confidential conversation. Also, there are no “right or wrong” answers. We are speaking with you because we want to learn about what it is like being a parent of a young child and what you think about behavioral health services for very young children. We are excited to be learning from you today and are thankful to you for taking the time to speak with us.

Do you have any questions? Are you ready to begin?

1. Introduction and warm up

**I would like to start with hearing a little bit about your children. You are a part of this research project because you are a parent of at least one child aged 5 or younger. How many children do you have aged 5 and under?**

**What are they like?**

*What do you love about your child[ren]? What do they do really well?*

*What about your child[ren] is challenging?*

**Now I would like you to tell me about yourself as a parent. What are you like as a parent?**

*What are your strengths as a parent? What do you do really well?*

*What challenges you as a parent?*

- **Follow up with what supports do you wish you had to improve this part of your parenting?**

*What about your parenting would you like to improve?*

**Thank you for telling me about your child[ren] and yourself as a parent. Now I want to move to some more general questions about children and their parents. So, I would like you to think about all parents and children and not just your own family for these next questions.**

1. Identifying a need for services for children

**What are some reasons or clues that an infant, that is a child ages birth to 12 months old, may need behavioral health services?**

*As needed:*

- *What does that look like to you?*
- *Tell me more about that.*
- *Describe what that might look like on a day-to-day basis.*

**What are some reasons or clues that a toddler, that is a child who is 2 or 3 years old, may need behavioral health services?**

*As needed:*

- *What does that look like to you?*
- *Tell me more about that.*
- *Describe what that might look like on a day-to-day basis.*

**What are some reasons or clues that a young child, ages 4 or 5 years old, may need behavioral health services?**

*As needed:*

- *What does that look like to you?*
- *Tell me more about that.*
- *Describe what that might look like on a day-to-day basis.*

1. Identifying a need for services for parents

**What are some reasons/clues that a parent of a young child may need support around parenting?**

*As needed:*

- *What does that look like to you?*
- *Tell me more about that.*
- *Describe what that might look like on a day-to-day basis.*

**What are some reasons/clues that a parent of a young children may need support for their own emotional wellbeing?**

*As needed:*

- *What does that look like to you?*
- *Tell me more about that.*
- *Describe what that might look like on a day-to-day basis.*

**To what degree does a parent’s wellbeing and mental health impact their child?**

*As needed:*

- *What does that look like to you?*
- *Tell me more about that.*
- *Describe what that might look like on a day-to-day basis.*

1. Ideal services

**For a young child with challenging behavior, what sorts of services or programs should be available to them?**

***Interviewer, try to get a sense about programs for children of different ages:*

- *What types of programs would be helpful for an infant?*
- *What types of programs would be helpful for a toddler?*
- *What types of programs would be helpful for a 4- or 5-year-old?*

*As needed:*

- *Walk me through that*
- *What does that look like?*
- *Tell me more about that*

**For a parent who needs support around *parenting*, what sorts of services or programs should be available to them?**

*As needed:*

- *Walk me through that*
- *What does that look like?*
- *Tell me more about that*

**For a parent who needs support around their own *wellbeing and mental health*, what sorts of services or programs should be available to them?**

*As needed:*

- *Walk me through that*
- *What does that look like?*
- *Tell me more about that*

1. Access to services

**What might make it hard for families to access or get behavioral healthcare for their child?**

*As needed:*

- When they mention barriers; **follow up with what does that look like/ what resolution is needed?**
- *How so/tell me more about that?*
- *Walk me through why “X” [use parents words for the barrier] might be a challenge.*
- *Describe what that might look like on a day-to-day basis.*

**What would help families get connected to behavioral health care for their child?**

*As needed:*

- *How so/tell me more about that?*
- *Walk me through why “X” [use parents words for the support] might be a challenge.*
- *Describe what that might look like on a day-to-day basis.*

**Sometimes families get connected to a behavioral health program or service for their child but miss their first appointment or never attend any appointments. What are your thoughts about that?**

*As needed:*

- *Walk me through that*
- *What does that look like?*
- *Tell me more about that*

**Once families are engaged in a program or service, which means attending regular appointments, what do you think might help families keep going and continue attending their appointments?**

*As needed:*

- *What does that look like to you?*
- *Tell me more about that.*
- *Describe what that might look like on a day-to-day basis.*

**How can healthcare providers, such as pediatricians, better involve parents in the referral process?**

**Thank you for sharing! I am going to move us along to the last part of the interview. Now that we have talked generally about child behavioral challenges and access to programs, I want to hear your thoughts about a specific parenting program for parents of young children with behavioral challenges called The Family Check-Up.**

1. Thoughts about the Family Check-Up

**Before I ask you about a specific parenting program, what have you experienced or heard of in terms of behavioral healthcare?**

**Have you heard of the Family Check-Up parenting program?**

********If yes:*

- *Where have you heard about it?*
- *Have you participated in the program?*
- *What was your experience like?*

*Then end with:* ***Thank you for sharing! Now I want to get your thoughts about our Family Check-Up informational video.***

****If no, jump to next prompt.*

**I am going to show you a brief informational video about the Family Check-Up parenting program. I would like you to imagine that you are a parent with a young child with behavioral challenges and this program is being offered to you. When we are done, I am going to ask a few questions about your thoughts about the video and then about the actual program.**

1. **First, I would like to hear what you thought about the video.**

*As needed:*

- *What stood out to you?*
- *What do you like about the video?*
- *What would you change about the video?*

1. **Now I would like to just hear what you thought about the program.**

*As needed:*

- *What stood out to you?*
- *What do you like about the program?*
- *What would you change about the program?*

1. **Imagining this program is being offered to you and your child, would you be interested in engaging in the program?**

*As needed:*

- *What about the video or the program itself makes you interested?*
- *What makes you less interested?*
- *What would need to change or be different for you to be interested?*
